# Cephalometric effects of the Jones Jig appliance followed by fixed appliances in Class II malocclusion treatment

**DOI:** 10.1590/2176-9451.19.3.044-051.oar

**Published:** 2014

**Authors:** Mayara Paim Patel, José Fernando Castanha Henriques, Karina Maria Salvatore de Freitas, Roberto Henrique da Costa Grec

**Affiliations:** 1 PhD in Orthodontics, School of Dentistry - University of São Paulo/Bauru.; 2 Full professor, University of São Paulo, USP.; 3 Adjunct professor, Masters program in Orthodontics, Ingá College, UNINGÁ.; 4 PhD resident in Orthodontics, School of Dentistry - University of São Paulo/Bauru.

**Keywords:** Malocclusion, Angle Class II, Corrective Orthodontics, Tooth movement

## Abstract

**Objective:**

The aim of this study was to cephalometrically assess the skeletal and dentoalveolar effects of Class II malocclusion treatment performed with the Jones Jig appliance followed by fixed appliances.

**Methods:**

The sample comprised 25 patients with Class II malocclusion treated with the Jones Jig appliance followed by fixed appliances, at a mean initial age of 12.90 years old. The mean time of the entire orthodontic treatment was 3.89 years. The distalization phase lasted for 0.85 years, after which the fixed appliance was used for 3.04 years. Cephalograms were used at initial (T_1_), post-distalization (T_2_) and final phases of treatment (T_3_). For intragroup comparison of the three phases evaluated, dependent ANOVA and Tukey tests were used.

**Results:**

Jones Jig appliance did not interfere in the maxillary and mandibular component and did not change maxillomandibular relationship. Jones Jig appliance promoted distalization of first molars with anchorage loss, mesialization and significant extrusion of first and second premolars, as well as a significant increase in anterior face height at the end of treatment. The majority of adverse effects that occur during intraoral distalization are subsequently corrected during corrective mechanics. Buccal inclination and protrusion of mandibular incisors were identified. By the end of treatment, correction of overjet and overbite was observed.

**Conclusions:**

Jones Jig appliance promoted distalization of first molars with anchorage loss represented by significant mesial movement and extrusion of first and second premolars, in addition to a significant increase in anterior face height.

## Abstract (pt)

**Objetivo:**

a proposta desse estudo foi avaliar cefalometricamente os efeitos esqueléticos e dentoalveolares do tratamento da má oclusão de Classe II com o distalizador Jones jig, seguido do uso do aparelho fixo corretivo.

**Métodos:**

a amostra constituiu de 25 pacientes com má oclusão de Classe II, tratados com o distalizador Jones Jig, seguido do uso de aparelho fixo corretivo, com média de idade inicial de 12,90 anos. O tempo médio de tratamento ortodôntico total foi de 3,89 anos. A fase de distalização durou 0,85 anos, e a fase de aparelho fixo pós-distalização foi de 3,04 anos. Foram utilizadas as telerradiografias nos tempos inicial (T_1_), pós-distalização (T_2_) e final do aparelho fixo (T_3_). Para comparação intragrupo nos três tempos avaliados, foram utilizados os testes ANOVA dependente e de Tukey.

**Resultados:**

os resultados demonstraram que o Jones Jig não interferiu no componente maxilar e mandibular, e não promoveu alterações na relação maxilomandibular. O Jones Jig promoveu distalização dos primeiros molares, com perda de ancoragem, mesialização e extrusão significativa dos primeiros e segundos pré-molares, e aumento significativo da altura facial anteroinferior ao final do tratamento. A maioria dos efeitos adversos ocorridos na fase de distalização intrabucal são posteriormente corrigidos durante a mecânica corretiva. Verificou-se vestibularização e protrusão dos incisivos inferiores. Ao final do tratamento, foi observada a correção dos trespasses horizontal e vertical.

**Conclusões:**

o distalizador Jones Jig promoveu a distalização dos primeiros molares, com perda de ancoragem, representada pela mesialização e extrusão significativa dos primeiros e segundos pré-molares, e aumento significativo da altura facial anteroinferior.

## INTRODUCTION

Class II malocclusion is an anteroposterior discrepancy characterized by dentoalveolar or skeletal change or a combination of both, of which mandibular retrusion is the predominant etiologic factor.^1^

There are several methods used to treat this anteroposterior discrepancy, in which case treatment is certainly diversified by patients' etiologic factor, growth pattern, age, degrees of cooperation and, specially, their chief complaint. They may opt for a treatment with or without extractions, with the use of headgear, intermaxillary elastics, functional or mechanical orthopedics removable appliances, fixed intraoral appliances, and even surgical-orthodontic treatment.

Patient's cooperation is a determinant factor for successful orthodontic treatment, thus, protocols that require minimal collaboration are of great value in orthodontic practice. Intraoral distalizers fulfill this function, i.e., they correct Class II malocclusion without entirely depending on the patient to achieve satisfactory results by the end of treatment.^2-5^

Intraoral distalizers differ in the site of action, either buccal or palatal, and promote different results during distalization.^6^ Another important factor is the type of anchorage which can be performed in deciduous molars or pre-molars, supported by two or four teeth.^7^ Anchorage reinforcement can be currently accomplished by miniscrews fixed on the palate, thereby promoting skeletal anchorage and reducing adverse effects that are characteristic of intraoral distalizers.^8^

However, intraoral distalization through intraoral fixed appliances is only the first phase of treatment that will be finalized with fixed corrective mechanics. In the literature, there are only a few studies scientifically assessing the results of the two treatment phases;^9-12^ most researches only assess the results of distalization.^2,4,7,13-17^ Therefore, it is of paramount importance to conduct a study that assesses distalization phase and post-distalization fixed appliance phase, separately.

Thus, the aim of this study was to cephalometrically assess the skeletal and dentoalveolar changes of young subjects with Class II malocclusion treated with the Jones Jig appliance followed by corrective fixed appliances, comparing the changes caused by the distalization phase with the changes of the corrective fixed appliances phase.

## MATERIAL AND METHODS

### Material

This study was approved by Ingá College Institutional Review Board. The prospective sample comprised 75 lateral cephalograms of 25 subjects treated with the Jones Jig appliance followed by fixed appliances.

The criteria for sample selection were based on the following characteristics: Presence of Class II, division 1 malocclusion; mild to moderate crowding; absence of previous orthodontic treatment; absence of supernumerary teeth or agenesis.

Fourteen out of 25 patients were male, while 11 were female, all presenting Class II, division 1 malocclusion, 4 full-cusp Class II, 3 3/4-cusp Class II, 7 1/2-cusp and 11 1/4-cusp Class II.

These patients were part of a prospective sample treated by a single student of Masters in Orthodontics at Ingá College. Class II molar relationship was initially corrected by means of the Jones Jig appliance and maintained as a result of the nightly use of headgear during the entire treatment. Fixed corrective appliances were also installed.

The mean initial age was 13.10 years (SD 1.40; minimum 10.83, maximum 16.24), the mean post-distalization age was 13.95 (SD 1.48; minimum 11.33, maximum 17.23), and the mean final age was 16.99 years (SD 1.87; minimum 15.03, maximum 23.15). The mean time of total orthodontic treatment was 3.89 years (SD 0.99). The distalization phase lasted for 0.85 years (SD 0.30; minimum 0.41, maximum 1.95) whereas the phase of fixed appliance after distalization lasted for 3.04 years (SD 0.97; minimum 1.73, maximum 5.93).

### Methods

#### Orthodontic treatment with Jones Jig appliance

The Jones Jig appliance^5^ was manufactured by Morelli^®^, and consisted of a 0.036-in steel body, a steel distal end of 0.016-in, a steel cursor on the mesial end and a stainless steel open spring which requires sequential activation. However, for this research, in order to dissipate light and continuous force, the steel spring was changed by a nickel-titanium spring of which mean size was 10 mm, GH manufacturer (Greenwood, USA & Canada). Distalizer installation started from the bandage of the maxillary second premolars in order to construct the modified Nance button. The compression spring corresponded to a distance of 5 mm, which promoted a dissipation of 120 grams (0.12N) of force, in average.

By the end of distalization and correction of molar relationship, an average overcorrection of 2 mm beyond normal molar relationship was endeavored. After removal of the Jones Jig, a modified Nance button was installed on the distalized molars in association with a headgear with middle-high traction (jeans helmet). This phase was followed by bonding of fixed orthodontic appliances (Morelli, Roth, slot 0.022 x 0.028-in). In the maxillary anterior retraction phase, the Nance button was removed and in addition to the night use of the headgear, (with a force of 250 grams, 0.25 N), 3/16-in Class II elastics were used, releasing an average force of 200 g/side (0.2 N), between 12 and 20 h/day. After the fixed orthodontic appliance was removed, a maxillary Hawley plate and a mandibular 3x3 were installed for retention.

#### Lateral cephalograms

Three lateral cephalograms of each patient were taken at three different times: T_1_ (initial), T_2_ (post-distalization) and T_3_ (final).

Each radiograph was traced with landmarks set in a darkened room. Through a Numonics A-30TL digitizing table, attached to an AMD K-6 II 500MHz microcomputer, the location of the cephalometric landmarks was transferred to the Dentofacial Planner 7.02 software (Dentofacial Planner Software Inc., Toronto, Ontario, Canada) in which measurements involving planes and lines were processed. Magnification factors were set at 6% and 9.8%.

#### Error of the method

Intra-examiner error was assessed by retracing and obtaining new measurements of 20 randomly selected cephalometric radiographs. The first and second measurements were performed within a month interval. The formula proposed by Dahlberg^18^ (Se^2^ = Sd^2^/2n) was applied to estimate the magnitude of casual errors, while systematic errors were analyzed by paired t-tests.^19^

#### Statistical analysis

Descriptive statistics was performed to obtain means and standard deviations of age and treatment times.

For intragroup comparisons of the three times evaluated, dependent ANOVA as well as Tukey tests were used whenever necessary.

STATISTICA for Windows (7.0 version, StatSoft.Inc.) software was used for analysis. Significance level was set at 5% (P < 0.05).

## RESULTS

Two systematic errors were observed (PTVI-A and NAP). Casual error ranged from 0.26 mm of overjet to 1.75 degrees of NAP variable.

There were significant changes in almost all components of the three assessed phases (Table 1). The Jones Jig appliance did not affect the maxillary and mandibular component and did not change maxillomandibular relationship. There was distalization of first molars with loss of anchorage, extrusion and mesial movement of first and second premolars. Anterior face height was increased after treatment. Mandibular incisors were uprighted and protruded. By the end of treatment, overjet and overbite were corrected (Table 1).

**Table 1:** Intragroup comparison of the three evaluated phases: Initial (T1), post-distalization (T2) and final (T3) (dependent ANOVA and Tukey tests).

Variables	T_1_	T_2_	T_3_	p
	Mean ± SD	Mean ± SD	Mean ± SD	
**Maxillary component**				
SNA (degrees)	81.91 ± 3.96^AB^	82.84 ± 4.20^A^	81.72 ± 4.84^B^	0.022*
Co-A (mm)	81.85 ± 5.21^A^	82.97 ± 5.65^A^	82.58 ± 5.48^A^	0.216
PTVI-A (mm)	47.85 ± 4.51^A^	48.06 ± 4.23^A^	48.77 ± 4.72^A^	0.148
**Mandibular component**				
SNB (degrees)	78.50 ± 3.00^A^	79.31 ± 3.26^A^	79.18 ± 4.37^A^	0.210
Co-Gn (mm)	104.41 ± 5.02^A^	106.30 ± 6.50^B^	109.67 ± 6.90^C^	0.000*
P-NB (mm)	1.40 ± 1.11^A^	1.47 ± 1.17^A^	1.66 ± 1.30^A^	0.284
PTVI-B (mm)	47.28 ± 5.43^A^	47.00 ± 5.11^A^	49.36 ± 5.98^B^	0.000*
**Maxillomandibular relationship**				
ANB (degrees)	3.42 ± 2.63^A^	3.57 ± 2.41^A^	2.54 ± 2.62^A^	0.055
NAP (degrees)	5.44 ± 5.89^A^	5.69 ± 5.77^A^	3.34 ± 5.58^A^	0.051
**Vertical component**				
FMA (degrees)	29.84 ± 4.17^A^	31.99 ± 4.88^B^	31.76 ± 4.04^B^	0.005*
SN.PP (degrees)	6.40 ± 4.07^A^	5.77 ± 3.70^A^	6.84 ± 3.34^A^	0.285
SN.GoGn (degrees)	31.80 ± 4.11^A^	31.86 ± 4.98^A^	32.25 ± 4.59^A^	0.650
SN.GoMe (degrees)	34.95 ± 4.18^A^	35.33 ± 4.46^A^	35.40 ± 4.63^A^	0.563
NS.Gn (degrees)	66.50 ± 3.58^A^	66.32 ± 3.89^A^	67.35 ± 4.34^A^	0.059
SN.Ocl (degrees)	9.77 ± 4.05^A^	10.04 ± 4.45^AB^	11.85 ± 3.40^B^	0.015*
LAFH (mm)	61.75 ± 5.54^A^	63.77 ± 5.71^B^	66.72 ± 6.95^C^	0.000*
**Maxillary dentoalveolar component**				
SN.1 (degrees)	107.26 ± 5.52^A^	111.77 ± 6.73^B^	106.16 ± 6.29^A^	0.000*
PTVI-1 (mm)	55.71 ± 5.23^A^	56.82 ± 5.06^A^	57.04 ± 5.35^A^	0.086
PP-1 (mm)	26.90 ± 2.82^A^	27.09 ± 2.95^A^	28.58 ± 3.38^B^	0.000*
1.NA (degrees)	25.33 ± 6.19^AB^	28.73 ± 6.93^A^	24.45 ± 6.95^B^	0.015*
1-NA (mm)	5.05 ± 2.72 ^A^	6.33 ± 2.67^A^	5.48 ± 3.36^A^	0.087
SN.4 (degrees)	82.20 ± 4.57^A^	94.60 ± 5.55^B^	80.66 ± 5.61^A^	0.000*
PTVI-4 (mm)	36.41 ± 3.87^A^	38.87 ± 3.61^B^	38.60 ± 4.21^B^	0.000*
PP-4 (mm)	19.20 ± 2.47^A^	20.61 ± 2.49^B^	21.20 ± 2.81^C^	0.000*
SN.5 (degrees)	78.24 ± 5.24^A^	88.78 ± 5.53^B^	79.80 ± 5.69^A^	0.000*
PTVI-5 (mm)	29.99 ± 3.76^A^	32.72 ± 3.73^B^	32.14 ± 4.33^B^	0.000*
PP-5 (mm)	18.69 ± 2.50^A^	20.64 ± 2.44^B^	20.69 ± 2.86^B^	0.000*
SN.6 (degrees)	65.48 ± 4.82^A^	55.30 ± 6.13^B^	66.80 ± 5.21^A^	0.000*
PTVI-6 (mm)	21.83 ± 3.61^A^	19.66 ± 3.34^B^	23.58 ± 4.23^C^	0.000*
PP-6 (mm)	16.90 ± 2.29^A^	16.46 ± 2.44^A^	19.15 ± 3.04^B^	0.000*
SN.7 (degrees)	50.26 ± 6.44^A^	48.57 ± 6.08^A^	55.42 ± 6.82^B^	0.000*
PTVI-7 (mm)	12.14 ± 3.14^A^	11.34 ± 3.36^A^	13.53 ± 3.98^B^	0.000*
PP-7 (mm)	11.48 ± 3.64^A^	11.82 ± 3.24^A^	15.68 ± 3.18^B^	0.000*
**Mandibular dentoalveolar component**				
1.NB degrees)	25.79 ± 5.70^A^	24.62 ± 5.75^A^	28.48 ± 5.17^B^	0.001*
1-NB (mm)	4.54 ± 2.21^A^	4.73 ± 2.04^A^	5.90 ± 1.93^B^	0.000*
PTVI-6i (mm)	20.86 ± 4.51^A^	21.04 ± 4.47^A^	23.25 ± 4.34 ^B^	0.000*
GoMe-6i (mm)	27.72 ± 2.78^A^	28.29 ± 2.76^A^	30.92 ± 3.62 ^B^	0.000*
**Dental relationships**				
Molar relationship (mm)	-0.35 ± 1.09^A^	-4.54 ± 1.07^B^	-2.76 ± 0.58^C^	0.000*
Overjet (mm)	4.59 ± 1.59^A^	5.71 ± 1.98^B^	2.80 ± 0.57^C^	0.000*
Overbite (mm)	3.82 ± 1.52^A^	3.40 ± 1.62^A^	2.35 ± 0.51^B^	0.000*

* statistically significant difference (p < 0.05)

- different letters mean statistically significant difference.

## DISCUSSION

The use of a control group could have added more data to this research by allowing the differentiation of changes produced by the Jones Jig and the fixed appliances from changes that occur with individuals' normal growth. However, the main objective of this study was to observe, separately, the changes in the period of use of the Jones Jig and in the period of use of fixed appliances, headgear and intermaxillary elastics. With this variation of time, there was no compatible control group that could have been used for comparison. Nevertheless, in no way, the lack of a control group invalidates the results obtained herein.

### Maxillary component

**Figure 1:**
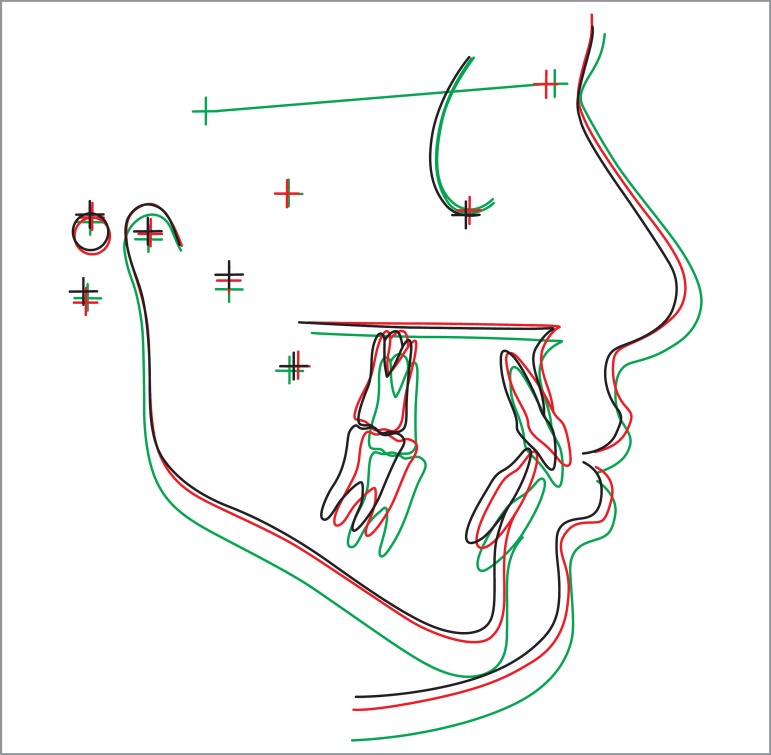
Mean cephalometric landmarks in the three observation phases: (T1, black; T2, red; and T3, green) (overlap on line SN).

Distalization with the Jones Jig followed by corrective fixed appliances did not produce statistically significant changes in the effective length of the maxilla (Fig 1, Table 1). This result was already expected, since intraoral distalizers do not promote skeletal changes, as reported in other studies.^9,14-16^

Nevertheless, there was significant maxillary retrusion.^9^ This change is probably related to the use of an extraoral headgear, since after distalization performed by means of the Jones Jig the headgear was used as anchorage so as to maintain molars distalized as well as to verticalize their roots.^9,20^

### Mandibular component

The effective length of the mandible was gradually and significantly increased, showing changes in the end of the distalization phase and at the end of corrective orthodontic treatment (Fig 1). Assessment of PTVI-B demonstrated statistically significant mandibular increase in the final phase of corrective treatment (Table 1). This change was probably related to craniofacial growth and development, which proves mandibular growth in the long-term.^21^

### Maxillomandibular relationship

Maxillomandibular relationship was improved by the end of corrective orthodontic treatment; however, these changes were not statistically significant in all three stages (Table 1). This result was already expected, specially at the end of distalization of maxillary molars, since intraoral distalizers do not significantly interfere in changes of bone bases.^9,14-16^

### Vertical component

The variables related to the vertical component showed a slight tendency towards angular increase in the phase of fixed corrective treatment; however, only FMA showed a statistically significant increase during distalization, a change that remained in the final stage. Likewise, the occlusal plane angle showed a statistically significant increase at the end of corrective treatment compared to the initial stage (Table 1). There was also a statistically significant increase in lower anterior face height in the post-distalization stage and at the end of corrective orthodontic treatment (Table 1).

Changes in cephalometric variables related to the vertical component certainly occurred due to clockwise rotation of the mandible probably caused by significant extrusion of first and second premolars during distalization.^9,16^

**Figure 2:**
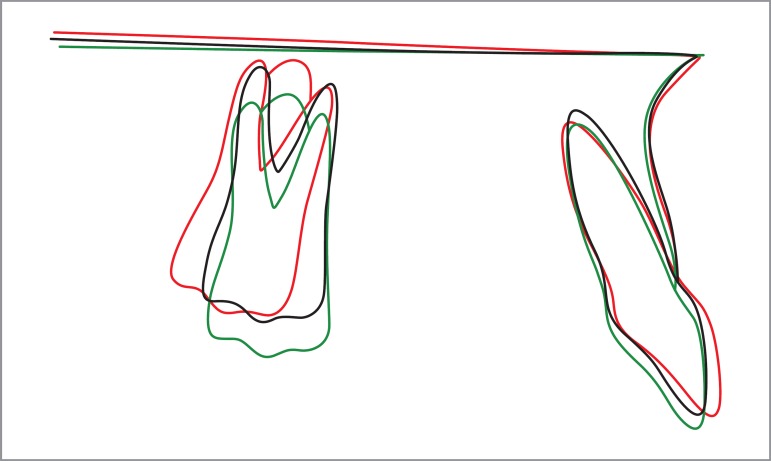
Maxillary teeth alterations in the three observation phases (T1, black; T2, red; and T3, green) (overlap on palatal plane).

Changes in the occlusal plane and LAFH were also observed at the end of corrective orthodontic treatment and were related to extrusion of first premolars and first and second molars (Fig 2). However, in this phase, vertical changes occurred to correct overbite, which decreased in 1.47 mm from the initial to the final stage.^9^ Moreover, increased LAFH may also be credited to normal vertical facial growth.

### Maxillary dentoalveolar component

#### Maxillary incisors

Maxillary incisors showed statistically significant buccal inclination in the post-distalization phase. This accentuated inclination is a characteristic of anchorage loss during intraoral distalization.^9,15-17^ However, in the final phase of orthodontic treatment, maxillary incisors showed significant palatal inclination, thereby reversing anchorage loss. Protrusion of maxillary incisors was not statistically significant (Table 1).

In the final phase of corrective orthodontic treatment, maxillary incisors also showed significant extrusion related to correction of buccal inclination, vertical displacement of the maxilla due to the craniofacial growth^22^ and the use of Class II elastics.

#### First and second maxillary premolars

In the post-distalization phase, first premolars demonstrated effects of anchorage loss related to distal movement through intraoral distalizers.^7,10-13,20^ There was significant linear and angular mesial movement associated with extrusion in relation to the palatal plane. Mesial angulation was corrected at the end of orthodontic treatment; however, mesial positioning remained in the final stage (Table 1), probably due to anterior displacement of the maxilla in an attempt to accompany mandibular displacement.^10,20^ First premolars showed significant extrusion at the end of distalization and orthodontic treatment.

Previous researches^9,16^ also assessed the effects of the Jones Jig appliance, but focused only on second premolars, which are the anchorage teeth selected for placement of the Nance button during intraoral distalization.

Second premolars showed similar behavior to first premolars, also characterizing anchorage loss due to intraoral distalization.^2,4,7,9-13,15-17,20,23,24^ There was statistically significant angular and linear mesial movement in the post-distalization phase. Conversely, in the final stage, mesial angulation was corrected, but mesial positioning also remained accompanying the displacement of the maxillary base in relation to the mandible.^10,20^

There was statistically significant extrusion during distalization in comparison to the initial position. This vertical change was related to mesial angulation and mesial movement, i.e., reflecting anchorage loss.^9,16^

Longitudinal observations show that extrusion, mesial angulation of premolars and anterior inclination of maxillary incisors are reversed effects that occur during active treatment with maxillary fixed appliances.^9,15^ They may even occur spontaneously in the period of verticalization and stabilization with the Nance button positioned in distalized molars. However, the vertical positioning of second premolars remained at the end of corrective treatment, similarly to the post-distalization phase. In other words, it is probable that the accentuated curve of Spee preserved extrusion of second premolars to correct overbite, which was greater at the beginning of treatment.

#### First and second maxillary molars

Maxillary first molars demonstrated significant distal angulation, as already anticipated in treatment of Class II with intraoral devices.^2-4,7,9,11-17,20^

Accentuated distal angulation is observed in all researches that assess distalization with the Jones Jig appliance,^5,9,14-17,25^ including cases of absolute anchorage.^26^ However, this angulation was reversed and, by the end of corrective orthodontic treatment, maxillary first molars showed a verticalized position in relation to the cranial base (Table 1 and Fig 2). This correction was already expected, since patients, by the end of the distalization process, used the extraoral headgear in order to anchor and verticalize distalized molars.^16,20^

Maxillary first molars were significantly distalized in the post-distalization phase; however, after corrective orthodontic treatment, significant angular and linear mesial movement was observed, probably due to correction of accentuated distal angulation and displacement of the maxilla of which goal is to maintain normal maxillomandibular relationship^10^ (Fig 2).

By the end of corrective orthodontic treatment, molar extrusion was statistically significant, probably due to correction of overbite (Fig 2). Extrusion of maxillary first molars can also be related to appositional maxillary growth and vertical flotation process.^22^ In this group, the use of headgear may not have promoted extrusion of first molars, since the headgear was used with medium-high traction (jeans helmet), thereby preventing tooth extrusion.

Maxillary second molars showed no statistically significant changes in the post-distalization phase; however, at the end of corrective orthodontic treatment, second molars showed mesial angulation in relation to the cranial base, as well as statistically significant mesialization. Statistically significant extrusion was also observed at the end of corrective orthodontic treatment. Changes caused to second molars were not only due to eruption, but also to movement of maxillary first molars in the corrective phase.^9^

In relation to maxillary first molars, second molars showed greater extrusion at the end of corrective treatment. This result can probably be attributed to the different stage of eruption of maxillary first and second molars.^9^

### Mandibular dentoalveolar component

#### Mandibular incisors

**Figure 3:**
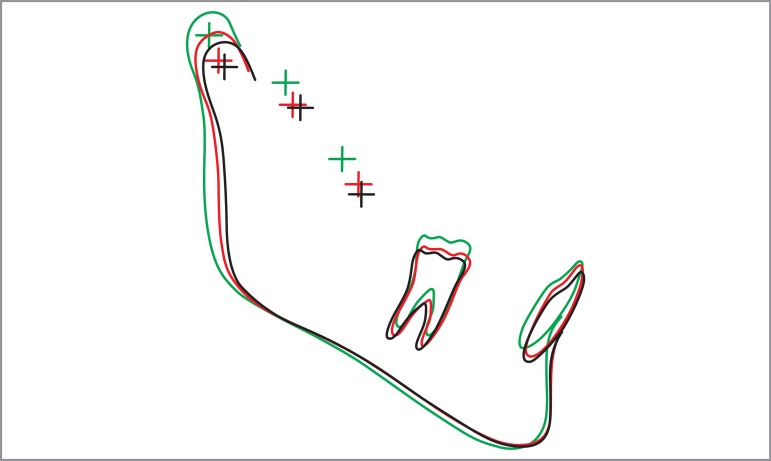
Manibular teeth alterations in the three observation phases (T1, black; T2, red; and T3, green) (overlap on mandibular plane).

With regard to mandibular incisors, there was statistically significant buccal inclination and protrusion at the end of corrective orthodontic treatment (Table 1 and Fig 3). This change was related to correction of overjet which was greater at the beginning of treatment. Therefore, changes observed in mandibular incisors occurred to decrease overjet and were due to the use of Class II elastics.

In the literature, only the study conducted by Brickman, Sinha and Nanda^9^ assessed the effect of Class II treatment with the Jones Jig appliance followed by corrective fixed appliances. Nevertheless, the authors did not report the behavior of mandibular incisors. However, studies that assessed other distalizers observed buccal inclination during orthodontic treatment.^10,11,20^ According to Angelieri et al,^20^ mandibular incisors are buccally inclined due to anterior mandibular displacement and the use of Class II elastics.

#### Mandibular first molars

Mandibular molars showed statistically significant mesialization during corrective treatment when initial and post-distalization phases were compared (Table 1). Class II molar relationship was corrected not only by maxillary molars distalization, but also by mesial movement of mandibular molars.^10,20^

As for the vertical component, at the end of corrective treatment, mandibular molars showed significant extrusion when the initial and post-distalization phases were compared (Fig 3). Changes in mandibular molars are not reported by studies assessing distalization with the Jones Jig ^5,15-17^ followed by fixed appliances;^9^ however, extrusion was observed in studies that use the Pendulum appliance.^10,11^ Therefore, it is assumed that mandibular molars extrusion may be related to the use of Class II elastics, vertical displacement due to appositional growth of the lower base of the mandible and to vertical floating.^22^

#### Dental relationships

Molar relationship showed statistically significant changes in the three phases of assessment. In the initial phase, patients showed Class II relationship, whereas in the post-distalization phase, there was overcorrection with molars ending up in a "super" Class I relationship, as suggested by previous studies.^9,11,12^ However, as expected, maxillary molars mesially moved during corrective treatment to establish normal molar relationship, (Table 1).

At the beginning of treatment, both overjet and overbite were increased. In the post-distalization phase, there was an increase in overjet due to buccal tipping of maxillary incisors, thereby characterizing anchorage loss related to distalization with the Jones Jig appliance.^16^ By the end of corrective orthodontic treatment, both overjet and overbite were reduced, thus demonstrating that orthodontic treatment goals were achieved. Correction of overjet was related to uprighting of maxillary incisors and buccal tipping of mandibular incisors, as described above. Correction of overbite, on the other hand, was probably related to extrusion of first and second premolars and first and second molars, as previously reported.

Brickman, Sinha and Nanda^9^ observed an increase of 0.45 mm in overjet and a decrease of 1.28 mm in overbite during distalization of maxillary molars. According to the authors, changes in overjet were related to mesial movement of maxillary premolars and buccal inclination of maxillary incisors, as reported by other studies assessing intraoral distalization.^12,13,16,20^

## CONCLUSION

The Jones Jig appliance did not interfere in maxillary and mandibular components and did not change maxillomandibular relationship. The Jones Jig appliance promoted distalization of first molars with anchorage loss, significant mesialization and extrusion of first and second premolars, and a significant increase in anterior face height at the end of treatment. Most adverse effects occurred during the intraoral distalization phase and were subsequently corrected with corrective mechanics. There was buccal inclination and protrusion of mandibular incisors. At the end of treatment, overjet and overbite correction was observed.
